# Developmental Assets Predictors of Life Satisfaction in Adolescents

**DOI:** 10.3389/fpsyg.2019.00236

**Published:** 2019-02-12

**Authors:** Ana Sofia Soares, José L. Pais-Ribeiro, Isabel Silva

**Affiliations:** ^1^Faculdade de Psicologia e Ciências da Educação, Universidade do Porto, Porto, Portugal; ^2^Faculdade de Ciências Humanas e Sociais, Universidade Fernando Pessoa do Porto, Porto, Portugal

**Keywords:** developmental assets, life satisfaction, positive youth development, positive developmental outcomes in adolescence, healthy development

## Abstract

The concept of Developmental Assets links positive features of developmental ecologies with personal skills, competences and values in order to further the understanding of optimal development. The purpose of this research was to explore the relationship between the experience of Developmental Assets and adolescent’s perception of Life Satisfaction. A convenience sample of 503 Portuguese students was evaluated, mean age of 15.92 years (*SD* = 1.17), 63% female gender. Findings revealed that both external and internal Developmental Assets are experienced differently across gender, grade and age. Results indicate that 74% of the adolescents report their Life Satisfaction to be in the positive range. Boys report significantly higher levels of Life Satisfaction than girls. Results show significant negative correlations between Life Satisfaction and age. The main effect of school grade on Life Satisfaction was not statistically significant. Findings revealed that 32 of the 40 Developmental Assets configured in the model showed a statistically significant positive relationship with Life Satisfaction. Also, results suggest that the greater the experience of Developmental Assets, the greater the Life Satisfaction. Stepwise regression was conducted to determine which Developmental Assets and demographic factors predict Life Satisfaction. Results suggest that the relationship between demographic variables and Life Satisfaction is weak, contributing modestly to the prediction of Life Satisfaction (2%). Nine Developmental Assets emerged as independent and significant predictors of Life Satisfaction: Overall Self-esteem made the largest contribution, with Family Support, Planning and decision Making, Sense of Purpose, Positive Family Communication, Positive Values of Caring, Youth as Resources, School Engagement and Other Adult Relationships also yield significant predictability. The total regression model (Developmental Assets and demographic variables) explained 54% of the variance in Life Satisfaction results. The present findings suggest the applicability and utility of the framework as a strategy to foster positive development in adolescence.

## Introduction

Emerging in the early 1990s, Positive Youth Development distinguish in the first half decade of the 21st century by shifting the focus from a deficit reduction and prevention paradigm to a strengths building approach, which aims at understanding what strengths, resources and experiences are important for successful youth development (Damon, 2004; Benson et al., 2006; Duncan et al., 2007; Lerner and Steinberg, 2009; Bonell et al., 2016).

Positive Youth Development represents a sea change in psychological theory and research (Damon, 2004) that focuses on: a kind of development that fosters positive outcomes; the nature of the youth (with emphasis on the inherent capacity of all youth for positive growth and development) and the identification of his/her developmental strengths (i.e., positive attitudes, skills, competences, and values that enhance healthy developmental trajectories); and developmental contexts, i.e., interactions with family, school, neighborhood, and societal contexts that provides support, opportunities, and resources (Benson et al., 2006).

In this field, increasing attention has been directed toward factors that benefit healthy youth development, which led to the emergence of models centering attention on the strengths, resources, and positive experiences of youth and their developmental contexts, seeking thus to conceptualize a positive development in adolescence (Eccles and Gootman, 2002; Damon, 2004; Hamilton et al., 2004; Small and Memmo, 2004; Lerner et al., 2006). Search Institute’s Developmental Assets framework constitutes one of this models. First posited in 1990, and refined in 1995 (Benson, 2006), the model was designed to give greater attention “to the positive developmental nutrients that young people need for successful development” (Benson et al., 2011, p.198). Developmental Assets are defined as a set of interrelated experiences, relationships, skills, and values suggested to enhance a broad range of positive youth outcomes and are assumed to operate similarly for all youth (Benson et al., 1998; Scales and Leffert, 2004; Benson, 2006; Benson et al., 2011; Sesma et al., 2013).

The framework of Developmental Assets is a theory-based model linking features of ecologies and context (External Assets) with personal skills, capacities, and values (Internal Assets), guided by the hypothesis that external and internal assets are dynamically interconnected “*Building Blocks*” (Benson et al., 2006, p.906), that, in combination, prevent health compromising behaviors (Benson and Scales, 2009) and enhance many forms of a successful development (i.e., *Thriving*) (Scales et al., 2000; Benson et al., 2006).

As shown in Table 1, the assets are grouped into domains of 20 External Assets (i.e., health-promoting features of the development) and 20 Internal Assets (i.e., skills, competencies, and positive values) which consist of four categories each. The External Assets comprise four categories: Support, Empowerment, Boundaries and Expectations, and Constructive Use of Time. The Internal Assets are also placed into four categories: Commitment to Learning, Positive Values, Social Competencies, and Positive Identity (for a detailed description, consult Benson et al., 1998, 2006, 2011; Scales and Leffert, 2004; Benson, 2006).

**Table 1:** The framework of 40 Developmental Assets^®^ for adolescents.

The framework of 40 Developmental Assets^®^ for adolescents	
External assets	Internal assets
Support category	Commitment to learning category
Asset 1: Family support	Asset 21: Achievement motivation
Asset 2: Positive family communication	Asset 22: School engagement
Asset 3: Other adult relationships	Asset 23: Homework
Asset 4: Caring neighborhood	Asset 24: Bonding to school
Asset 5. Caring school climate	Asset 25: Reading for pleasure
Asset 6: Parent involvement in schooling	Positive values category
Empowerment category	Asset 26: Caring
Asset 7: Community values youth	Asset 27: Equality and social justice
Asset 8: Youth as resources	Asset 28: Integrity
Asset 9: Service to others	Asset 29: Honesty
Asset 10: Safety	Asset 30: Responsibility
Boundaries and expectations category	Asset 31: Restraint
Asset 11: Family boundaries	Social competencies category
Asset 12: School boundaries	Asset 32: Planning and decision making
Asset 13: Neighborhood boundaries	Asset 33: Interpersonal competence
Asset 14: Adult role models	Asset 34: Cultural competence
Asset 15: Positive peer influence	Asset 35: Resistance skills
Asset 16: High expectations	Asset 36: Peaceful conflict resolution
Constructive use of time category	Positive identity category
Asset 17: Creative activities	Asset 37: Personal power
Asset 18: Youth programs	Asset 38: Self-esteem
Asset 19: Religious community	Asset 39: Sense of purpose
Asset 20: Time at home	Asset 40: Positive view of personal future

*Copyright© 2012, Search Institute, Minneapolis, MN; 800-888-7828; www.search-institute.org. All rights reserved. Do not reproduce*.

Research suggests a relationship between Developmental Assets and a range of positive youth developmental outcomes, indicating that *all* young people benefit from the experiences, opportunities, and personal characteristics that are captured in both External and Internal Assets. The most consistent finding in this regard is that the Developmental Assets framework appears to have comparable validity across young people’s gender, race/ethnicity and socioeconomic background (Benson et al., 1998, 2006; Leffert et al., 1998; Scales et al., 2000, 2013; Taylor et al., 2005; Benson, 2006; Filbert and Flynn, 2010). Evidence also suggests that some Developmental Assets are more relevant for some demographic groups than for others. Findings revealed that both External and Internal Assets are expressed or experienced differently across gender, grade, and age. Also, results suggest a decline in self-reported assets across adolescence (Benson et al., 1998; Leffert et al., 1998; Scales et al., 2000).

One of the central assumptions of the Developmental Assets model is that assets are additive or cumulative. Research indicates that the more Developmental Assets young people experience, the better off they tend to be, across a range of academic, psychological, social-emotional, and behavioral indicators of well-being (Benson et al., 2006, 2011). The consequence of this additive function is the reverse of that found in the research on risk factors: the cumulative impact of risk factors, i.e., negative outcomes increases additively or exponentially as the number of risk factors increases (Rutter, 1987; Esbensen et al., 2009). The cumulative-risk framework has been extended to the cumulative-assets framework, which was adopted to focus on youth positive outcomes. Benson et al. (2011, p.204) explain that “a developmental asset is an agent or characteristic of the individual or his/her developmental ecologies (…) that is related to the increased probability of positive outcomes,” which “increase additively or exponentially as the number of developmental assets increases.” A cumulative effect was consistently noted, indicating that a higher number of both external and internal assets is negatively associated with risk behaviors (Benson et al., 1998; Leffert et al., 1998; Atkins et al., 2002; Aspy et al., 2010; Chew et al., 2010; Cheney et al., 2015) and positively associated with positive developmental outcomes, such as academic achievement and subjective well-being (Benson et al., 1998; Scales et al., 2000, 2006; Taylor et al., 2005; Valois et al., 2009; Filbert and Flynn, 2010; Oman et al., 2015).

The number of Developmental Assets youth experience has considerable implications for their health and well-being, regardless of the outcome under study. Although, research also suggests that not every outcome is affected, similarly by exactly the same assets, indicating that specific assets, or clusters of Assets, best predict concurrent and longitudinal outcomes, with the assets varying depending on the outcome in question (Scales et al., 2000; Leffert et al., 1998; Taylor et al., 2005; Theokas et al., 2005; Aspy et al., 2010; Chew et al., 2010; Filbert and Flynn, 2010; Cheney et al., 2015; Oman et al., 2015).

Within the Positive Youth Developmental field, Life Satisfaction is suggested as an important indicator of positive development (Park, 2004). Among the constituent components of Subjective Well-Being, which is conceptualized as multifaceted in nature, with both affective and cognitive components (Diener et al., 1999), Life Satisfaction constitutes a distinct construct representing a cognitive and global evaluation of the quality of one’s life as a whole (Pavot and Diener, 1993). Life Satisfaction refers to a judgmental process in which individuals assess the quality of their lives on the basis of their own unique set of criteria (Shin and Johnson, 1978). In other words, it consists in a conscious cognitive judgment of one’s own life in which the criteria for judgment depend on the person (Pavot and Diener, 1993).

Therefore, Life Satisfaction constitutes a cognitive component of Subjective Well-Being playing an important role in positive development. Subjective Well-Being represents a key indicator of positive development, as well as a broad enabling factor that enhances health outcomes (Park, 2004). Similar to a higher experience of Developmental Assets, higher levels of Life Satisfaction in adolescents are related to good adaptation and optimal mental health (see Proctor et al., 2009, for a review).

However, a review of literature showed few studies addressing the relationship between perceived Life Satisfaction and Developmental Assets in the context of Positive Youth Development approach. Research indicate a significant positive relationship between increased experience of Developmental Assets and perception of Life Satisfaction (Valois et al., 2009; Oberle et al., 2011; Zullig et al., 2011; Oman et al., 2015). In addition, research highlights the importance of exploring this relationship in different age groups, since one study indicates that in college students not all Developmental Assets are related to higher Satisfaction with Life (Zullig et al., 2011). Also, prospective associations among youth Assets and successful transition into early adulthood suggest that young adults who possessed a higher number of Assets in their youth were more likely to report better transition into early adulthood (Oman et al., 2015). Thus, it is relevant to understand how each of these important areas relates to another given the implications for the promotion of a positive development in adolescence.

Each of the Developmental Assets identified in the framework is important for a healthy development. Well-Being and positive development can be best understood when adopting an integrative model that considers both individual and contextual Assets in relation to developmental outcomes of adolescents.

The purpose of this research was to explore the relationship between the experience of Developmental Assets and adolescent’s perception of Life Satisfaction, and to specifically explore which Developmental Assets and demographic variables were predictive of adolescent Life Satisfaction.

## Materials and Methods

### Study Design and Participants

An observational cross-sectional study was conducted over a convenience sample of 503 Portuguese students, in grades 9–12th in a public school (general education) located in the Northern part of Portugal, in the district of Braga, one of the most populous districts in the country. Portugal is one of the European Union Countries and the educational system is like the other European partners. Our adolescent population share the same characteristics of European Community population. If we consider the United Nations Human Development Report, Portugal is placed in the Very High Human Development group. Participants were mostly from low-class and middle-class families, 63% female gender, and ranged from 13 to 19 years of age (*M* = 15.92; *SD* = 1.17).

### Developmental Assets

Developmental Assets were measured using the Portuguese-European version of the Search Institute Profiles of Student Life: Attitudes and Behaviors survey – A&B (Soares et al., unpublished), originally developed by the Search Institute (Benson et al., 1998; Leffert et al., 1998; Search Institute, 2018). The 156-item survey includes measures of each of the 40 developmental assets as well as other constructs, including thriving indicators, high-risk behaviors and standard demographic questions. For the present study, 92 items measuring the 40 developmental assets were utilized, along with demographic items. Regarding Asset items, most are measured on a 5-point Likert scale ranging from, for example, 1 = strongly agree to 5 = strongly disagree; 1 = not important to 5 = extremely important; and 1 = not at all like me to 5 = very much like me. There are several approaches to describe the state of developmental assets among adolescents. Assets can be scored as continuous variables (with higher scores designating more assets). This approach examines the standardized continuous distributions, i.e., with Likert-type response options, of youth measured on the Assets.

Also, for communication purposes, the Assets can be reported as binary variables. Assets scored on a binary basis (a youth has or does not have the asset) yield an index of 0–40 binaries, which describes the average number of Assets each youth possesses, as well as the percentage of youth who possess each of the Developmental Assets. In addition, it yields the concept of Levels of Assets, which is useful to articulate the broader continuum of healthy development and to define four Levels of Assets based on how many of the Assets an adolescent possesses (the sample is divided into quartiles based on the total number of Assets), representing a continuum from “at-risk” to “optimal” development: Level One - *At-risk Development*: 0 to 10 Assets; Level Two – *Vulnerable Development*: 11 to 20 Assets; Level Three – *Adequate Development*: 21 to 30 Assets; and Level Four – *Optimal* Development: 31 to 40 Assets.

Portuguese version of A&B adopts a conservative perspective, with the purpose of maintaining a conceptual and metric equivalence that reflects the theoretical assumptions established by the model. The process of cross-cultural adaptation followed the recommendations of International Test Commission (2005) and Wild et al. (2005). Data were inspected using what Cronbach and Meehl (1955) define *nomological network*. The adoption of the unitary perspective of validity can be considered the central and fundamental element of any evaluation technique (Kane, 2001, 2013; Elosua and Iliescu, 2012; Pais-Ribeiro, 2013). Validity is, by definition, “the degree to which evidence and theory support the interpretation of test scores for proposed uses of tests” (American Educational Research Association et al., 2014, p.11).

Thus, the Portuguese version of A&B has similar qualities in terms of validity to those of original version. Twenty-one of the 40 Developmental Assets are measured by at least 3 items, and most of those have acceptable reliability ranging from 0.60 to the 0.80s (Cronbach, 1951). Thirteen of the Assets are measured with single items, therefore, internal consistency does not apply. The reliability coefficients of the categories are: Support α = 0.81; Empowerment α = 0.76; Boundaries and Expectations α = 0.65; Constructive Use of Time.39 (note, is a multidimensional category); Commitment to Learning α = 0.67; Positive Values α = 0.78; Social Competencies α = 0.72; Positive Identity α = 0.83 (Soares et al., unpublished).

### Life Satisfaction

Life Satisfaction was assessed using the Portuguese-European version of the Satisfaction with Life Scale – SWLS (Neto, 1993), originally developed by Diener et al. (1985). SWLS is a five-item instrument that assesses global Life Satisfaction, on a 7-point Likert scale ranging from 1 = strongly disagree to 7 = strongly agree. Scores on the SWLS are used as continuous variables, with higher scores designating more satisfaction (range of the scale from 5 to 35). In addition, scores on the SWLS can be interpreted in terms of absolute as well as relative Life Satisfaction (Diener et al., 1985; Pavot and Diener, 1993).

A score of 20 represents the neutral point on the scale, the point in which the respondent is equally satisfied as dissatisfied. A score between 21 and 25 represents *Slightly Satisfied*, between 26 and 30 represents *Satisfied* and between 31 and 35 represents *Extremely Satisfied*. Also, a score between 15 and 19 represents *Slightly Dissatisfied*, between10 and 14 represents *Dissatisfied*, and between 5 and 9 *Extremely Dissatisfied*. Neto (1993) noted that reliability figures of the Portuguese version of the scale were found to be favorable. In the present study the reliability coefficient of the scale was found to be acceptable, α = 0.83 (Cronbach, 1951).

### Procedure

This study was approved by Direção-Geral da Educação (Portuguese Directorate-General for Education, process number 0416200002). Ethics approval for this research was obtained from the Ethics Committee of the Direção-Geral da Educação. This study was also carried out in accordance with regulations concerning professional ethics as stated in Ordem dos Psicólogos Portugueses (Portuguese Psychologists Association) (Código Deontológico da Ordem dos Psicólogos Portugueses, 2016) and obeying the same recommendations established by World Medical Association’s Declaration of Helsinki. Comissão Nacional de Proteção de Dados (National Commission for Data Protection, process number: 3154/2014) confirmed the anonymity of participants in data collection. Parents/guardians of all students who participated in the study gave written informed consent for data collection, use, and publication of the results. Data collection was conducted in the school. The survey was administered anonymously in classroom setting with standardized instructions. Students who received parental/guardian consent were asked to complete the survey. Participants place the survey in a sealed envelope. This study ensured the anonymity and confidentiality of the data and their unique and exclusive use for research purposes.

### Data Analysis

#### Developmental Assets

The Assets are measured by one or more survey items with a minimum of five Likert-type response options. In a first analysis, these items are combined and then convened into a binary variable of each Asset. This analysis allows to assess the percentage of youth who possessed each of the 40 Developmental Assets. Also, this binary format yield an index, of 0–40 binaries, describing the average number of Assets each youth possesses, which is analyzed by gender, grade and total sample: *t* test was performed in order to determine gender difference on mean of Assets; analysis of variance (one-way ANOVA, Post-Hoc Bonferroni) was conducted to determine the effect of school grade on mean of Developmental Assets; Pearson’s correlation was conducted in order to investigate the relation between the mean of Developmental Assets and age. In addition, this binary format yield the concept o Level of Assets which define four Levels of Assets based on how many of the Assets an adolescent possesses (the sample is divided into quartiles based on the total number of Assets): 0–10; 11–20; 21–30; and 31–40; this analysis allows to assess the percentage of students falling within each of the four Levels.

To analyze the experience of each of the 40 individual Assets we examined the standardized continuous distributions (i.e., with all possible Likert-type response options) of youth measured on the assets: *t* tests were performed to assess differences across gender in the experience of each of the 40 Assets; analyzes of variance (one-way ANOVA, Post-Hoc Bonferroni) were conducted to determine the effect of school grade on the experience of Assets; Pearson’s correlations were conducted in order to investigate the relation between the experience of Assets and age.

#### Life Satisfaction

In order to analyze the experience of Life Satisfaction we examined the standardized continuous distributions (i.e., with Likert-type response options) and the following analyses were performed: descriptive statistics, including means and standard deviations, by gender, grade and total sample; *t* test was performed to assess differences across gender in Life Satisfaction; analysis of variance (one-way ANOVA, Post-Hoc Bonferroni) was performed to determine the effect of school grade on Life Satisfaction; Pearson’s correlation was performed in order to investigate the relation between Life Satisfaction and age.

#### Relationship Between Developmental Assets and Life Satisfaction

To determine the cumulative impact of the Assets experience on Life Satisfaction, analysis of variance (one-way ANOVA, Post-Hoc Bonferroni) was conducted. Pearson’s correlations were performed in order to assess the relation between Life Satisfaction and the experience of each of the 40 Developmental Assets. Stepwise regression was conducted to determine which Developmental Assets and demographic factors predict Life Satisfaction. The Stepwise regression analysis conducted in this study includes two sets of predictor variables: the first set includes only the demographic measures (gender, age and school grade) and the second contains (in addition to the first set) all the 40 Developmental Assets as predictor variables. There were no missing data in the data set. Analyses were carried out using SPSS version 25.

## Results

### Developmental Assets

Results indicate that the mean of Assets based on an index of 0–40 binaries is 19.60 (*SD* = 6.08). Girls (*M* = 19.54; *SD* = 6.12) and boys (*M* = 19.72; *SD* = 6.00) did not differ statistically on mean of Assets, *t*(462) = -0.312, *p* = 0.755. Concerning grades, means (with standard deviations in parentheses) for 9th–12th grades are: 21.07 (6.23), 19.34 (6.03), 19.08 (6.13), 18.99 (5.67), respectively. Analysis of variance (one-way ANOVA) was performed, and results indicate that the main effect of school grade was statistically significant, *F*(3,463) = 2.68: results suggest that the lower the school grade is, the greater the average experience of Assets. Regarding age, results show significant negative correlations between mean of Developmental Assets and age, *r*(468) = -0.11, *p* = 0.020, and they suggest that greater experience of Developmental Assets is associated with lower age.

Table 2 presents the percentage of adolescents possessing each of the binary Assets by gender, grade and total sample. Regarding the concept of Levels of Assets, the percentages of students falling within each of the four Levels of Assets were distributed as follows: 6 % (*n* = 28) have 0 to 10 Assets (Level One: At-risk Development), 51% (*n* = 238) have 11 to 20 Assets (Level Two: Vulnerable Development), 40% (*n* = 186) have 21 to 30 Assets (Level Three: Adequate Development) and 4% (*n* = 16) have 31 to 40 Assets (Level Four: Optimal Development).

**Table 2:** Experience of developmental assets by gender, grade, and total sample in percentage.

	9th Grade (*n* = 110)			10th Grade (*n* = 176)			11th Grade (*n* = 139)			12th Grade (*n* = 77)		
Developmental assets	% with asset			% with asset			% with asset			% with asset		
	M	F	T	M	F	T	M	F	T	M	F	T
1 Family support	91	82	87	82	76	78	80	74	76	59	66	64
2 Positive family communication	66	49	57	50	47	48	53	58	56	19	48	38
3 Other adult relationships	56	39	48	42	39	40	48	47	47	40	37	38
4 Caring neighborhood	30	40	37	34	31	32	27	29	28	37	28	32
5 Caring school climate	29	37	33	31	26	28	30	23	25	30	16	21
6 Parent involvement schooling	37	37	37	32	27	29	18	26	23	11	16	15
7 Community values youth	41	40	40	36	33	34	33	45	41	22	33	29
8 Youth as resources	39	31	35	36	28	31	40	29	33	19	23	21
9 Service to others	30	24	28	24	21	22	20	18	20	15	34	27
10 Safety	64	57	60	71	38	49	76	48	57	78	46	57
11 Family boundaries	46	38	41	39	34	36	56	27	36	15	26	22
12 School boundaries	58	47	53	39	41	40	38	35	35	45	34	38
13 Neighborhood boundaries	41	42	43	57	48	51	40	35	37	41	34	37
14 Adult role models	31	38	34	32	30	31	18	35	29	16	41	32
15 Positive peer influence	79	86	82	71	83	79	58	74	69	54	64	61
16 High expectations	66	73	70	50	52	52	50	47	49	48	43	45
17 Creative activities	16	24	20	13	9	10	12	13	12	22	18	20
18 Youth programs	59	24	41	44	27	33	51	26	35	45	48	47
19 Religious community	30	43	38	34	41	38	29	33	32	22	34	30
20 Time at home	90	96	93	90	92	92	87	94	91	74	82	79
21 Achievement motivation	64	77	69	68	79	75	71	73	73	71	73	71
22 School engagement	75	84	79	87	85	86	85	78	80	63	76	72
23 Homework	24	38	31	42	61	54	13	46	35	45	71	61
24 Bonding to school	52	71	62	45	63	57	47	50	49	48	52	51
25 Reading for pleasure	6	27	17	16	23	21	9	20	16	11	18	16
26 Caring	71	69	71	60	72	68	56	64	61	63	82	75
27 Equality, social justice	85	91	87	73	92	85	78	95	89	89	90	90
28 Integrity	81	77	78	81	90	87	76	87	84	85	92	90
29 Honesty	87	93	90	78	89	85	87	95	92	74	100	91
30 Responsibility	83	95	89	79	89	85	78	88	84	85	92	90
31 Restraint	33	38	35	23	17	19	7	12	10	4	4	4
32 Planning, decision-making	51	38	47	42	34	37	42	37	38	41	38	39
33 Interpersonal competence	53	55	54	34	44	40	42	53	49	33	56	48
34 Cultural competence	49	35	42	42	53	49	47	52	50	30	52	44
35 Resistance skills	49	33	40	44	46	45	51	52	51	33	46	42
36 Peaceful conflict resolution	43	63	57	48	58	54	49	60	57	62	74	70
37 Personal power	37	26	30	32	26	29	36	32	33	33	32	33
38. Self-esteem	67	33	49	55	28	38	53	38	43	63	38	47
39 Sense of purpose	58	33	45	58	32	41	50	46	47	56	40	46
40 Positive view of personal future	58	57	57	45	29	35	56	39	44	45	42	43

*M, male adolescent; F, female adolescent; T, total sample*.

Analysis of standardized continuous distributions of Developmental Assets, performing *t* tests, showed statistically significant differences across gender in the experience of the 40 Assets. Girls report significantly higher experience of Developmental Assets of Positive Peer Influence, *t*(494) = 2.92, *p* < 0.05, Religious Community, *t*(493) = 3.31, *p* < 0.0001, Achievement Motivation, *t*(497) = 3.75, *p* < 0.0001, School Engagement, *t*(496) = 2.17, *p* < 0.05, Homework, *t*(491) = 6.9, *p* < 0.0001, Reading for Pleasure, *t*(495) = 4.55, *p* < 0.0001, Positive Values of Caring, *t*(497) = 3.84, *p* < 0.0001, Equality and Social Justice, *t*(497) = 5.01, *p* < 0.0001, Integrity, *t*(497) = 3.16, *p* < 0.05, Honesty, *t*(497) = 3.75, *p* < 0.0001, Responsibility, *t*(497) = 3.94, *p* < 0.0001, Interpersonal Competence, *t*(496) = 3.04, *p* < 0.05, and Peaceful Conflict Resolution, *t*(490) = 2.72, *p* < 0.05. Boys report significantly higher experience of the following Developmental Assets: Youth as Resources, *t*(497) = 2.31, *p* < 0.05, Safety, *t*(496) = 4.91, *p* < 0.0001, Youth Programs, *t*(495) = 3.85, *p* < 0.0001, Personal Power, *t*(497) = 2.34, *p* < 0.05, Self-esteem, *t*(497) = 6.18, *p* < 0.0001, Sense of Purpose, *t*(496) = 5.68, *p* < 0.0001, and Positive View of Personal Future, *t*(497) = 2.76, *p* < 0.05. No other significant differences were established among genders concerning Developmental Assets.

Analysis of variance (one-way ANOVA, Post-Hoc Bonferroni) was conducted to determine the effect of school grade on the experience of Developmental Assets. Significant differences were found for the experience of the following Developmental Assets: Family Support, *F*(3,498) = 3.97, *p* < 0.0001 (9th > 12th), Caring School Climate, *F*(3,498) = 3.12, *p* < 0.05 (10th > 12th), Parent Involvement in Schooling, *F*(3,496) = 11.59, *p* < 0.0001 (9th > 11th; 9th > 12th), Service to Others, *F*(3,495) = 2.58, *p* < 0.05 (12th > 10th), Family Boundaries, *F*(3,498) = 4.59, *p* < 0.05 (9th > 12th; 10th > 12th), School Boundaries, *F*(3,498) = 4.36, *p* < 0.05 (9th > 11th; 9th > 12th), Positive Peer Influence, *F*(3,495) = 5.22, *p* < 0.0001 (9th > 12th; 10th > 12th), High Expectations, *F*(3,495) = 8.90, *p* < 0.0001 (9th > 10th; 9th > 11th; 9th > 12th), Youth Programs, *F*(3,496) = 2.64, *p* = < .05 (12th > 11th), Time at Home, *F*(3,494) = 5.17, *p* < 0.0001 (10th > 12th; 11th > 12th), School Engagement, *F*(3,492) = 2.80, *p* < 0.05 (10th > 12th), Homework, *F*(3,492) = 12.32, *p* < 0.0001 (10th > 9th; 12th > 9th; 10th > 11th; 12th > 11th), positive values of Restraint, *F*(3,498) = 24.27, *p* < 0.0001 (9th > 10th; 9th > 11th; 9th > 12th; 10th > 11th; 10th > 12th) and Positive View of Personal Future, *F*(3,498) = 4.02, *p* < 0.05 (9th > 10th). No other main effect of school grade was significant.

Results also show significant correlations between the experience of Assets and age. Results indicate significant negative correlations between age and the experience of the following Developmental Assets: Family Support, *r*(503) = -0.16, *p* < 0.0001, Caring Neighborhood, *r*(501) = -0.11, *p* < 0.05, Caring School Climate, *r*(503) = -0.10, *p* < 0.05, Parent Involvement in Schooling, *r*(501) = -0.21, *p* < 0.0001, Family Boundaries, *r*(503) = -0.11, *p* < 0.05, Positive Peer Influence, *r*(500) = -0.21, *p* < 0.0001, Higher Expectations, *r*(500) = -0.18, *p* < 0.0001, Time at Home, *r*(499) = -0.21, *p* < 0.0001, Achievement Motivation, *r*(503) = -0.09, *p* < 0.05, School Engagement, *r*(502) = -0.15, *p* < 0.001, Bonding to School, *r*(503) = -0.10, *p < 0*.05, positive values of Restraint, *r*(503) = -0.34, *p* < 0.0001. Results suggest that the younger the age, the greater the experience of these Developmental Assets. On the other hand, results indicate significant positive correlations between age and the experience of the following Developmental Assets: Service to Others, *r*(500) = 0.16, *p* < 0.0001, Youth Programs *r*(501) = 0.09, *p* < 0.05, and Positive Values of Integrity, *r*(503) = 0.10, *p <*0.05, and suggest that the higher the age, the greater the experience of this Developmental Assets. No other significant association was established among these variables.

### Life Satisfaction

Table 3 presents the means of Life Satisfaction (with standard deviations in parentheses) by gender, grade and total sample. Concerning gender, boys report significantly higher levels of Life Satisfaction than girls, *t*(495) = 2.63, *p* < 0.05. Analysis of variance (one-way ANOVA) was performed, and results suggest that the main effect of school grade on Life Satisfaction was not statistically significant *F*(3,496) = 2.07, *p* = 0.104. Results show significant negative correlations between Life Satisfaction and age, *r*(501) = -0.13, *p* < 0.05, and suggest that the lower the age is, the greater the Life Satisfaction.

**Table 3:** Means (standard deviations in parenthesis) for scores on the satisfaction with life scale by gender, grade, and total sample.

		Life satisfaction				
Gender		Grade				Total sample
Male (*n* = 184) *M* (*SD*)	Female (*n* = 313) *M* (*SD*)	9th Grade (*n* = 109) *M* (*SD*)	10th Grade (*n* = 175) *M* (*SD*)	11th Grade (*n* = 139) *M* (*SD*)	12th Grade (*n* = 77) *M* (*SD*)	(*n* = 500) *M* (*SD*)
25.69 (6.03)	24.20 (6.16)	25.93 (6.40)	24.20 (5.93)	24.77 (5.94)	24.14 (6.43)	24.74 (6.14)

Scores on the SWLS, interpreted in terms of absolute as well as relative life satisfaction (Pavot and Diener, 1993), reveal that most students report being satisfied with their lives. Twenty three percent (*n* = 116) report their life satisfaction to be in the range of *Slightly Satisfied*, 34% (*n* = 172) *Satisfied*, and 17% (*n* = 84) *Extremely Satisfied*. However, 14% (*n* = 72) fall in the range *Slightly Dissatisfied*, 5% (*n* = 27) *Dissatisfied*, and 1% (*n* = 7) *Extremely Dissatisfied.* Five percent (*n* = 23) report to be *Equally Satisfied as Dissatisfied.*

### Developmental Assets and Life Satisfaction

Table 4 presents the means of Life Satisfaction reported (with standard deviations in parentheses) by Assets Level. To determine the cumulative impact of the Assets on Life Satisfaction, analysis of variance (one-way ANOVA, Post-Hoc Bonferroni) was conducted. There was a significant effect of Asset Level, *F*(3,462) = 53.49, *p* < 0.0001 (Post-Hoc Bonferroni indicated that the groups all differed significantly from one another), such that adolescents with higher Assets Levels are more likely to report higher Life Satisfaction.

**Table 4:** Means (standard deviations in parenthesis) for scores on the satisfaction with life scale by levels of assets.

	Levels of assets					
	Level 1 0–10 (*n* = 28) *M* (*SD*)	Level 2 11–20 (*n* = 237) *M* (*SD*)	Level 3 21–30 (*n* = 185) *M* (*SD*)	Level 4 31–40 (*n* = 16) *M* (*SD*)	*F*	*p*
Life satisfaction	17.96 (5.90)	22.97 (5.66)	27.64 (4.85)	32.19 (1.72)	53.49	<0.0001

Significant positive correlations were found between Life Satisfaction and the experience of Developmental Assets: 32 of the 40 Assets configured in the model showed a statistically significant positive relationship with Life Satisfaction. Results indicate that the greater Life Satisfaction is, the greater the experience of Developmental Assets. Significant positive correlations were found between Life Satisfaction and the experience of the following Developmental Assets: Family Support, *r*(501) = 0.49, *p* < 0.0001, Positive Family Communication, *r*(501) = 0.42, *p* < 0.0001, Other Adult Relationships, *r*(495) = 0.31, *p* < 0.0001, Caring Neighborhood, *r*(499) = 0.24, *p* < 0.0001, Caring School Climate, *r*(501) = 0.28, *p* < 0.0001, Parent Involvement in Schooling, *r*(499) = 0.27, *p* < 0.0001, Community Values Youth, *r*(501) = 0.45, *p* < 0.0001, Youth as Resources, *r*(501) = 0.46, *p* < 0.0001, Safety, *r*(500) = 0.19, *p* < 0.0001, Family Boundaries, *r*(501) = 0.24, *p* < 0.0001, School Boundaries, *r*(501) = 0.17, *p* < 0.0001, Adult Role Models, *r*(498) = 0.30, *p* < 0.0001, Positive Peer Influence, *r*(498) = 0.16, *p* < 0.0001, High Expectations, *r*(498) = 0.31, *p* < 0.0001, Youth Programs, *r*(499) = 0.11, *p* < 0.05, Achievement Motivation, *r*(501) = 0.23, *p* < 0.0001, School Engagement, *r*(500) = 0.28, *p* < 0.0001, Homework, *r*(495) = 0.11, *p* < 0.05, Bonding to School, *r*(501) = 0.18, *p* < 0.0001, positive values of Caring, *r*(501) = 0.17, *p* < 0.0001, Equality and Social Justice, *r*(501) = 0.13, *p < 0*.05, Integrity, *r*(501) = 0.09, *p* < 0.05, Responsibility, *r*(501) = 0.24, *p* < 0.0001, and Restraint, *r*(501) = 0.17, *p* < 0.0001, Planning and Decision Making, *r*(500) = 0.37, *p* < 0.0001, Interpersonal Competence, *r*(500) = 0.20, *p* < 0.0001, Cultural Competence, *r*(500) = 0.13, *p* < 0.05, Resistance Skills, *r*(500) = 0.22, *p* < 0.0001, Personal Power, *r*(501) = 0.45, *p* < 0.0001, Self-esteem, *r*(501) = 0.58, *p* < 0.0001, Sense of Purpose, *r*(500) = 0.50, *p* < 0.0001, and Positive View of Personal Future, *r*(501) = 0.38, *p* < 0.0001. One exception was noted: results indicate significant negative correlations between Life Satisfaction and the experience of the Developmental Asset of Reading for Pleasure, *r*(501) = -0.09, *p* < 0.05, and they suggest that the higher the number of hours spent reading for pleasure (not as part of school work), the lower the Satisfaction with Life.

Stepwise regression was conducted to examine the extent to which Developmental Assets and demographic factors predict Life Satisfaction. The regression analysis conducted include two sets of predictor variables. The first set includes only the demographic measures (gender, age, and school grade) and the second contains (in addition to the first set) all the 40 Developmental Assets (Table 5). Two demographic variables emerged as independent and significant predictors of Life Satisfaction: gender (male) and age (lower). These two factors accounted for 2% of the variance in Life Satisfaction results. In addition, nine Developmental Assets emerged as independent and significant predictors of Life Satisfaction: overall Self-esteem made the largest contribution to the prediction of Life Satisfaction, with Family Support, Planning and Decision Making, Sense of Purpose, Positive Family Communication, Positive Values of Caring, Youth as Resources, School Engagement, and Other Adult Relationships also yield significant predictability. These eleven variables accounted for 54% of the variance in Life Satisfaction results, *R*^2^ = 0.55, *R*^2^(Adj.) = 0.54, *F*(11,450) = 49.24, *p* < 0.0001.

**Table 5:** Predictors of life satisfaction (stepwise regression analysis).

		Life satisfaction			
	Predictor	β	*T*	Δ*R*^2^	*R*^2^(Adj)
1	Gender	0.00	0.06	0.02	0.01
2	Age	-0.05	-1.57	0.01	0.02
3	Self-esteem	0.29	6.39***	0.34	0.36
4	Family support	0.08	1.81	0.07	0.43
5	Planning and decision making	0.12	3.43**	0.03	0.46
6	Sense of purpose	0.19	4.42***	0.02	0.49
7	Positive family communication	0.14	3.59***	0.02	0.50
8	Positive values: caring	0.12	3.36**	0.02	0.52
9	Youth as resources	0.10	2.38*	0.01	0.53
10	School engagement	0.09	2.76*	0.01	0.53
11	Other adult relationships	0.08	2.21*	0.00	0.54

^∗^*p < 0.05, ^∗∗^*p* < 0.01, and ^∗∗∗^*p* < 0.001*.

## Discussion

From an overall perspective, the results from this study suggest that very few Portuguese adolescents experience enough Assets: on average adolescents tend to experience half of the 40 Developmental Assets. These results are similar to those presented by Benson et al. (1998) in an aggregate sample of American students. Girls and boys did not differ statistically in the average number of Assets. Also, results suggest that the lower the school grade is, as so the lower the age is, the greater the average experience of Assets. Previous studies show a similar pattern (Benson et al., 1998; Leffert et al., 1998; Benson, 2006), suggesting that the number of Assets tend to decline through middle school and then begins to stabilize during high school. Adolescence is a period of adjustment, characterized by a universal process of separation and individuation, necessary to lay the foundation for successful transition to adulthood. So, the average decline in the experience of Assets observed in previous studies may in part reflect this grow-and-differentiation process (Benson, 2006).

In this regard, Benson (2006) notes that *asset-building* efforts may not prevent some decline in Assets as young people move into adolescence, as adolescence is – and should be – a time of significant change. The goal, then, should focus on reducing the level of decline in Assets early in adolescence, foster an earlier “*bottoming out*” of reported Assets, and facilitate an earlier and larger recovery of level of Assets by late adolescence (Benson, 2006, p. 65).

However, it is important to note that youth-development efforts should focus on sustaining the positive strengths present in a young person’s life and building upon them Benson (2006).Taking into account the importance of this suggestion, the results from this study also suggest that some Developmental Assets are more relevant for some demographic groups than for others. Findings from this study suggest that both External and Internal Assets are expressed or experienced differently across gender, grade and age. These results are consistent with other studies (Benson et al., 1998; Leffert et al., 1998; Scales et al., 2000; Benson, 2006). It should be noted that these two types of results found in this study, that represent aspects of similarity and diversity in the experience of Assets, allow the identification of Assets that are important to *all* adolescents, while indicate the relevance of specific Assets in adolescents with different characteristics. This knowledge provides the development of strategies that are culturally relevant, gender and age appropriate, sensitive to contextual influences, particular characteristics and needs of adolescents. In turn, this variety of relationships suggest something distinctive about how some adolescents experience or express Assets, indicating the importance of considering these differences identified when focus on promoting the well-being of adolescents.

Results indicate that adolescents report their Life Satisfaction to be on the positive range (74% of the students), similar to findings of previous studies (Diener et al., 1985; Neto, 1993; Pavot and Diener, 1993; Huebner et al., 2000; Proctor et al., 2009). The mean of Life Satisfaction falls in the range of *Slightly Satisfied*, which is comparable to corresponding figures reported by previous studies (Diener et al., 1985; Neto, 1993; Pavot and Diener, 1993). Data analysis suggests that boys have higher Life Satisfaction than girls. Results suggest that the main effect of school grade on Life Satisfaction was not significant. This finding suggest that the experience of Life Satisfaction may not be contextually influenced by school grade. However, results indicate a significant negative correlation between Life Satisfaction and age, suggesting that the lower the age, the greater Life Satisfaction. This finding is similarly, supported by international research (Proctor et al., 2009) suggesting that global Life Satisfaction tends to decline slightly with the onset and progression of adolescence.

The results of this study provide evidence for the additive nature of Developmental Assets in relation to their impact on positive developmental outcomes among adolescents, and extend knowledge specifically to the results of Life Satisfaction. These findings suggest that the higher the number of Developmental Assets that an adolescent reports, the more likely he or she will be to also report higher Life Satisfaction. We must also note, taking into account the mean Life Satisfaction reported by Asset Level, that results indicate that the mean on the Satisfaction With Life Scale falls in the *Slightly Dissatisfied* range in the group *At-risk Development*, in the *Slightly Satisfied* range in the group *Vulnerable Development*, in the *Satisfied* range in the group *Adequate Development*, and in the *Extremely Satisfied* range in the group *Optimal Development*. To our knowledge, this is the first study examining this particular match. From an overall perspective, the findings concerning the association between Levels of Assets and Life Satisfaction support the assumption of the cumulative-assets framework suggesting that a higher number of external and internal Assets promotes adolescent positive development.

In line with the study’s predictions, Life Satisfaction was positively associated with the majority of the Developmental Assets. Results indicate that greater Life Satisfaction is associated with greater experience of Developmental Assets. One exception was noted in the experience of the asset Reading for Pleasure, suggesting that the higher the number of hours spent reading for pleasure (not as part of school work), the lower the Satisfaction with Life reported.

Results suggest that the relationship between demographic variables and Life Satisfaction is weak, contributing only modestly to the prediction of youth Life Satisfaction, consistent with other reports (Diener, 1984; Gilman and Huebner, 2003; Diener and Diener, 2009; Proctor et al., 2009). Results demonstrate the impact of the experience of Developmental Assets on the prediction of Life Satisfaction: the Developmental Assets accounted for a considerable amount of the variance of Life Satisfaction. Both Internal and External Assets contributed unique variance to Life Satisfaction results, with Internal Assets being strongly predictive of Life Satisfaction.

The strongest predictor of Life Satisfaction was the Internal Asset of Self-esteem, in the Positive Identity category, a finding that is also in line with previous studies (Diener, 1984; Diener and Diener, 2009). In the same category, Sense of Purpose also contributes to the prediction of youth Life Satisfaction. In particular, related to Internal Assets, results highlight the importance of Social Competencies involving the personal skill of Making Plans and Decisions. Also important to note, the Positive Value of Caring, which indicates that adolescents places high value on helping other people, and which was found to be a predictor of Life Satisfaction. School Engagement, reflecting commitment to learning, was, in addition, a meaningful Internal Asset predictor of Life Satisfaction.

In turn, External Assets provide the setting conditions or supportive environment to optimize Life Satisfaction, and also contribute to the prediction of youth Life Satisfaction. In the category of Support, the Assets related to family context, namely, Family Support and Positive Family Communication, as well as Support from Non-parent Adults appeared to be predictors of Life Satisfaction. One interesting result concerns one of the Assets related to the Empowerment category, namely, Youth as Resources. Our findings highlight the importance of empowering young people to such an extent that they feel valued, for them to feel that others view them as resources and support the importance of providing opportunities for her or him to contribute in meaningful ways to society.

### Study Limitations and Future Research

There are limitations in this study that should be kept in mind when considering the results. First, the data are cross-sectional and thus Developmental Asset/Life Satisfaction causality cannot be inferred. Results suggest the importance of longitudinal and experimental studies to determine whether Life Satisfaction is a consequence or a determinant of possessing Developmental Assets. Furthermore, measures in the present study are self-reported. Future studies should include triangulation of measurement of external Assets or setting conditions from other reporters such as parents or teachers.

## Conclusion

The Developmental Assets framework, which focuses on a Positive Youth Development approach, provides a holistic perspective that promotes a successful development in several psychological, physical and/or socio-emotional developmental domains, and includes key contexts of adolescent development, such as family, school and community, and contributes to the understanding of several positive developmental outcomes such as Life Satisfaction.

Research suggests that the sheer number of Developmental Assets youth experience has considerable implications for their health and well-being, regardless of the outcome that is targeted. But research also indicates that every outcome is not affected similarly by exactly the same Assets. Rather, in addition to the accumulation hypothesis, we also found in the results of this study, that specific Assets are especially influential predictors of Life Satisfaction.

These study findings extend knowledge on the nature of developmental experiences and resources in relation to their impact on positive developmental outcomes among adolescents, providing knowledge on how to foster Life Satisfaction in adolescence. By adopting Developmental Assets framework we suggest the likely efficacy of a dual strategy of both attempting to build all 40 assets throughout young people’s ecologies and especially targeting the promotion of specific clusters of assets to enhance Life Satisfaction.

These study results suggest the applicability and utility of the framework as a strategy to foster positive development in adolescence.

## Author Contributions

This manuscript stems from a study developed from a ongoing Ph.D. project of AS at the University of Porto, under the supervision of JP-R and IS who are authors of this manuscript.

## Conflict of Interest Statement

The authors declare that the research was conducted in the absence of any commercial or financial relationships that could be construed as a potential conflict of interest.

## References

[B1] American Educational Research Association American Psychological Association National Council on Measurement in Education (2014). *Standards for Educational and Psychological Testing.* Washington, DC: American Educational Research Association.

[B2] Aspy C. B. Vesely S. K. Tolma E. L. Oman R. F. Rodine S. Marshall L. (2010). Youth assets and delayed coitarche across developmental age groups. *J. Early Adolesc.* 30 277–304. 10.1177/0272431609333297

[B3] Atkins L. A. Oman R. F. Vesely S. K. Aspy C. B. McLeroy K. (2002). Adolescent tobacco use: the protective effects of developmental assets. *Am. J. Health Promot.* 16 198–205. 10.4278/0890-1171-16.4.198 11913325

[B4] Benson P. L. (2006). *All Kids are our Kids: What Communities must do to Raise Caring and Responsible Children and Adolescents.* San Francisco, CA: Jossey-Bass.

[B5] Benson P. L. Leffert N. Scales P. C. Blyth D. A. (1998). Beyond the “village” rhetoric: creating healthy communities for children and adolescents. *Appl. Dev. Sci.* 2 138–159. 10.1207/s1532480xads0203_3

[B6] Benson P. L. Scales P. C. (2009). Positive youth development and the prevention of youth aggression and violence. *Int. J. Dev. Sci.* 3 218–234. 10.3233/DEV-2009-3302

[B7] Benson P. L. Scales P. C. Hamilton S. F. Sesma A. Jr. (2006). “Positive youth development: theory, research and applications,” in *Handbook of Child Psychology, Theoretical Models of Human Development*, eds Damon W. Lerner R. M. (New York, NY: John Wiley), 894–941.

[B8] Benson P. L. Scales P. C. Syvertsen A. K. (2011). “The contribution of the developmental assets framework to positive youth development theory and practice,” in *Advances in Child Development and Behavior*, eds Lerner R. M. Lerner J. V. Benson J. B. (Amsterdam: Elsevier), 197–230. 10.1016/b978-0-12-386492-5.00008-7 23259193

[B9] Bonell C. Hinds K. Dickson K. Thomas J. Fletcher A. Murphy S. (2016). What is positive youth development and how might it reduce substance use and violence? A systematic review and synthesis of theoretical literature. *BMC Public Health* 16:135. 10.1186/s12889-016-2817-3 26864336 PMC4748512

[B10] Cheney M. K. Oman R. F. Vesely S. K. (2015). Prospective associations among youth assets in young adults and tobacco use. *Am. J. Prev. Med.* 48 S94–S101. 10.1016/j.amepre.2014.09.021 25528715

[B11] Chew W. Osseck J. Raygor D. Eldridge-Houser J. Cox C. (2010). Developmental assets: profile of youth in a juvenile justice facility. *J. Sch. Health* 80 66–72. 10.1111/j.1746-1561.2009.00467.x 20236404

[B12] Código Deontológico da Ordem dos Psicólogos Portugueses. (2016). *Código Deontológico da Ordem dos Psicólogos Portugueses.* Available at: www.ordemdospsicologos.pt/ficheiros/documentos/web_cod_deontologico_pt_revisao_2016.pdf [accessed September 1 2018].

[B13] Cronbach L. J. (1951). Coefficient alpha and the internal structure of tests. *Psychometrika* 16 297–334. 10.1007/BF02310555

[B14] Cronbach L. J. Meehl P. E. (1955). Construct validity in psychological tests. *Psychol. Bull.* 52 281–302. 10.1037/h0040957 13245896

[B15] Damon W. (2004). What is positive youth development? *Ann. Am. Acad. Pol. Soc. Sci*. 591 13–24. 10.1177/0002716203260092

[B16] Diener E. Diener M. (2009). “Cross-cultural correlates of life satisfaction and self-esteem,” in *Culture and Well-Being. Social Indicators Research Series*, ed. Diener E. (Dordrecht: Springer), 71–91. 10.1007/978-90-481-2352-0_4

[B17] Diener E. Emmons R. A. Larsen R. J. Griffin S. (1985). The satisfaction with life scale. *J. Pers. Assess.* 49 71–75. 10.1207/s15327752jpa4901_13 16367493

[B18] Diener E. (1984). Subjective well-being. *Psychol. Bull.* 95 542–575. 10.1037/0033-2909.95.3.542 6399758

[B19] Diener E. Suh E. M. Lucas R. E. Smith H. L. (1999). Subjective well-being: three decades of progress. *Psychol. Bull.* 125 276–302. 10.1037/0033-2909.125.2.276

[B20] Duncan P. M. Garcia A. C. Frankowski B. L. Carey P. A. Kallock E. A. Dixon R. D. (2007). Inspiring healthy adolescent choices: a rationale for and guide to strength promotion in primary care. *J. Adolesc. Health* 41 525–535. 10.1016/j.jadohealth.2007.05.024 18023780

[B21] Eccles J. S. Gootman J. A. (2002). *Community Programs to Promote Youth Development.* Washington, DC: National Academy Press.

[B22] Elosua P. Iliescu D. (2012). Tests in Europe: where we are and where we should go. *Int. J. Test.* 12 157–175. 10.1080/15305058.2012.657316

[B23] Esbensen F. A. Peterson D. Taylor T. J. Freng A. (2009). Similarities and differences in risk factors for violent offending and gang membership. *Aust. N. Z. J. Criminol.* 42 310–335. 10.1375/acri.42.3.310

[B24] Filbert K. M. Flynn R. J. (2010). Developmental and cultural assets and resilient outcomes in first nations young people in care: an initial test of an explanatory model. *Child Youth Serv. Rev.* 32 560–564. 10.1016/j.childyouth.2009.12.002

[B25] Gilman R. Huebner S. (2003). A review of life satisfaction research with children and adolescents. *Sch. Psychol. Rev.* 18 192–205. 10.1521/scpq.18.2.192.21858

[B26] Hamilton S. F. Hamilton M. A. Pittman K. (2004). “Principles for youth development,” in *The Youth Development Handbook: Coming of Age in American Communities*, eds Hamilton S. F. Hamilton H. M. A. (Thousand Oaks, CA: Sage), 3–22. 10.4135/9781452232560.n1

[B27] Huebner E. S. Drane J. W. Valois R. F. (2000). Levels and demographic correlates of adolescent life satisfaction reports. *Sch. Psychol. Int.* 21 281–292. 10.1177/0143034300213005

[B28] International Test Commission. (2005). *International Guidelines on Test Adaptation.* Available at: https://www.intestcom.org [accessed September 1 2018].

[B29] Kane M. (2001). Current concerns in validity theory. *J. Educ. Meas.* 38 319–334. 10.1111/j.1745-3984.2001.tb01130.x

[B30] Kane M. (2013). Validating the interpretations and uses of test scores. *J. Educ. Meas.* 50 1–73. 10.1111/jedm.12000

[B31] Leffert N. Benson P. L. Scales P. C. Sharma A. R. Drake D. R. Blyth D. A. (1998). Developmental assets: measurement and prediction of risk behaviors among adolescents. *Appl. Dev. Sci.* 2 209–230. 10.1207/s1532480xads0204_4

[B32] Lerner R. M. Alberts A. E. Jelicic H. Smith L. M. (2006). “Young people are resources to be developed: promoting positive youth development through adult-youth relations and community assets,” in *Mobilizing Adults for Positive Youth Development*, eds Clary E. G. Rhodes J. E. (New York, NY: Springer), 19–39.

[B33] Lerner R. M. Steinberg L. (2009). “The scientific study of adolescent development,” in *Handbook of Adolescent Psychology*, eds Lerner R. M. Steinberg L. (Hoboken, NJ: John Wiley & Sons, Inc), 3–14. 10.1002/9780470479193

[B34] Neto F. (1993). The satisfaction with life scale: psychometrics properties in an adolescent sample. *J. Youth Adolesc.* 22 125–134. 10.1007/BF01536648

[B35] Oberle E. Schonert-Reichl K. A. Zumbo B. D. (2011). Life satisfaction in early adolescence: personal, neighborhood, school, family, and peer influences. *J. Youth Adolesc.* 40 889–901. 10.1007/s10964-010-9599-1 21042841

[B36] Oman R. F. Vesely S. K. Aspy C. B. Tolma E. L. (2015). Prospective associations among assets and successful transition to early adulthood. *Am. J. Public Health* 105 51–56. 10.2105/AJPH.2014.302310 25393188 PMC4265912

[B37] Pais-Ribeiro J. (2013). Medida na avaliação psicológica. *Psicol. Saúde Doenças.* 14 245–263.

[B38] Park N. (2004). The role of subjective well-being in positive youth development. *Ann. Am. Acad. Pol. Soc. Sci.* 591 25–39. 10.1177/0002716203260078

[B39] Pavot W. Diener E. (1993). Review of the satisfaction with life scale. *Psychol. Assess.* 5 164–172. 10.1037/1040-3590.5.2.164

[B40] Proctor C. L. Linley P. A. Maltby J. (2009). Youth life satisfaction: a review of the literature. *J. Happiness Stud.* 10 583–630. 10.1007/s10902-008-9110-9

[B41] Rutter M. (1987). Psychosocial resilience and protective mechanisms. *Am. J. Orthopsychiatry* 57 316–331. 10.1111/j.1939-0025.1987.tb03541.x 3303954

[B42] Scales P. C. Benson P. L. Dershem L. Fraher K. Makonnen R. Nazneen S. (2013). Building developmental assets to empower adolescent girls in rural Bangladesh: evaluation of project Kishoree Kontha. *J. Res. Adolesc.* 23 171–184. 10.1111/j.1532-7795.2012.00805.x

[B43] Scales P. C. Benson P. L. Leffert N. Blyth D. A. (2000). Contribution of developmental assets to the prediction of thriving among adolescents. *Appl. Dev. Sci.* 4 27–46. 10.1207/S1532480XADS0401_3

[B44] Scales P. C. Benson P. L. Roehlkepartain E. C. Sesma A. van Dulmen M. (2006). The role of developmental assets in predicting academic achievement: a longitudinal study. *J. Adolesc.* 29 691–708. 10.1016/j.adolescence.2005.09.001 16274739

[B45] Scales P. C. Leffert N. (2004). *Developmental Assets: A Synthesis of the Scientific Research on Adolescent Development.* Minneapolis, MN: Search Institute.

[B46] Search Institute. (2018). *User Guide for The Attitudes & Behaviors Survey.* Available at: http://www.search-institute.org/sites/default/files/a/A&B-Survey-User-Guide.pdf [accessed September 1 2018].

[B47] Sesma A. Jr. Mannes M. Scales P. C. (2013). “Positive adaptation, resilience and the developmental assets framework,” in *Handbook of Resilience in Children*, eds Goldstein S. Brooks R. B. (New York, NY: Springer), 427–442.

[B48] Shin D. C. Johnson D. M. (1978). Avowed happiness as an overall assessment of the quality of life. *Soc. Indic. Res.* 5 475–492. 10.1007/BF00352944

[B49] Small S. Memmo M. (2004). Contemporary models of youth development and problem prevention: toward an integration of terms, concepts, and models. *Fam. Relat.* 53 3–11. 10.1111/j.1741-3729.2004.00002.x

[B50] Taylor C. S. Smith P. R. Taylor V. A. von Eye A. Lerner R. M. Balsano A. B. (2005). Individual and ecological assets and thriving among African American adolescent male gang and community-based organization members: a report from wave 3 of the “overcoming the odds” study. *J. Early Adolesc.* 25 72–93. 10.1177/0272431604271771

[B51] Theokas C. Almerigi J. B. Lerner R. M. Dowling E. M. Benson P. L. Scales P. C. (2005). Conceptualizing and modeling individual and ecological asset components of thriving in early adolescence. *J. Early Adolesc.* 25 113–143. 10.1177/0272431604272460

[B52] Valois R. F. Zullig K. J. Huebner E. S. Drane J. W. (2009). Youth developmental assets and perceived life satisfaction: is there a relationship? *Appl. Res. Qual. Life* 4:315 10.1007/s11482-009-9083-9

[B53] Wild D. Grove A. Martin M. Eremenco S. McElroy S. Verjee-Lorenz A. (2005). Principles of good practice for the translation and cultural adaptation process for patient-reported outcomes (PRO) measures: report of the ISPOR task force for translating adaptation. *Value Health* 2 94–104. 10.1111/j.1524-4733.2005.04054.x 15804318

[B54] Zullig K. J. Teoli D. A. Ward R. M. (2011). Not all developmental assets are related to positive health outcomes in college students. *Health Qual. Life Outcomes* 9:52. 10.1186/1477-7525-9-52 21752293 PMC3155895

